# Long-Term Serial Follow-Up of Pulmonary Artery Size and Wall Shear Stress in Fontan Patients

**DOI:** 10.1007/s00246-015-1326-y

**Published:** 2016-01-12

**Authors:** Sjoerd S. M. Bossers, Merih Cibis, Livia Kapusta, Wouter V. Potters, Miranda M. Snoeren, Jolanda J. Wentzel, Adriaan Moelker, Willem A. Helbing

**Affiliations:** Division of Cardiology, Department of Pediatrics, Erasmus Medical Centre, Sophia Children’s Hospital, Sp-2429, PO Box 2060, 3000 CB Rotterdam, The Netherlands; Department of Radiology, Erasmus Medical Centre, Rotterdam, The Netherlands; Division of Cardiology, Department of Biomedical Engineering, Erasmus Medical Centre, Rotterdam, The Netherlands; Department of Pediatric Cardiology, Radboud University Medical Centre, Nijmegen, The Netherlands; Department of Pediatrics, Pediatric Cardiology Unit, Tel-Aviv Sourasky Medical Centre, Tel Aviv, Israel; Department of Radiology, Amsterdam Academic Medical Center, Amsterdam, The Netherlands; Department of Radiology, Radboud University Medical Centre, Nijmegen, The Netherlands

**Keywords:** Fontan, TCPC, Pulmonary arteries, Shear stress, Cardiac magnetic resonance imaging

## Abstract

Pulmonary arterial (PA) flow is abnormal after the Fontan operation and is marked by a lack of pulsatility. We assessed the effects of this abnormal flow on the size and function of the PA’s in Fontan patients in long-term serial follow-up. Twenty-three Fontan patients with serial follow-up were included. Median age was 11.1 (9.5–16.0) years at baseline and 15.5 (12.5–22.7) years at follow-up. Median follow-up duration was 4.4 (4.0–5.8) years. Flow and size of the left pulmonary artery were determined using phase-contrast MRI. From this wall shear stress (WSS), distensibility and pulsatility were determined. A group of healthy peers was included for reference. Flow and pulsatility were significantly lower in patients than in controls (*p* < 0.001). Mean area was comparable in patients and controls, but distensibility was significantly higher in controls (*p* < 0.001). Mean and peak WSS were significantly lower in Fontan patients (*p* < 0.001). Between baseline and follow-up, there was a significant increase in normalized flow (15.1 (14.3–19.1) to 18.7 (14.0–22.6) ml/s/m^2^, *p* = 0.023). Area, pulsatility, distensibility and WSS did not change, but there was a trend toward a lower mean WSS (*p* = 0.068). Multivariable regression analysis showed that flow, area and age were important predictors for WSS. WSS in Fontan patients is decreased compared to healthy controls and tends to decrease further with age. Pulsatility and distensibility are significantly lower compared to healthy controls. Pulmonary artery size, however, is not significantly different from healthy controls and long-term growth after Fontan operation is proportionate to body size.

## Introduction

The Fontan circulation, used as a final palliative approach for univentricular heart defects, is marked by a direct connection of the systemic venous return to the pulmonary arteries (PA) [17]. This leads to passive pulmonary blood flow that is no longer driven by a subpulmonary ventricle. As a result, these children have an abnormal flow pattern in their PAs early on in life, characterized by a nearly complete loss of pulsatility [13, 26].

It is hypothesized that this abnormal flow and pulsatility could influence PA growth and function on the long term. Since the Fontan circulation is dependent on a low transpulmonary pressure gradient, it is important to monitor the development of these vessels. Not only is longitudinal data on PA size and function in the Fontan circulation scarce, but studies investigating PA size in Fontan patients have also shown conflicting results [24, 26, 32].

Wall shear stress (WSS) is important in the development of vasculature [7]. Reduced levels of WSS have been related to PA endothelial dysfunction [9]. There is an inverse relation between vessel diameter and WSS [6]. A previous study from our center has shown that WSS is reduced in Fontan patients during rest, and also during exercise [26].

To date, only few studies have investigated PA growth using MRI long term after the Fontan operation. No studies have measured the course of WSS over time in Fontan patients.

The objective of this study was to assess PA growth, function and WSS over time in Fontan patients using MRI.

## Methods

### Patients

All patients participated in an ongoing cross-sectional multicenter study in the Netherlands. Patients who underwent Fontan operation at young age, either according to an atriopulmonary connection or a total cavopulmonary connection (TCPC), were included. Patients with mental retardation and contraindications for cardiac magnetic resonance imaging (CMR) were excluded. Informed consent was obtained from all patients and/or their parents. The study was approved by the institutional review boards of the participating centers. The study was conducted in accordance with the Helsinki Declaration. Medical records were reviewed for anatomical and operative details.

### MRI Imaging

In an earlier study by our group, we showed that accurate flow measurement in the RPA was not feasible in every patient because of the short distance between the connection of the superior caval vein and the first branching point of the RPA. Comparison of LPA and RPA flow in the aforementioned study did not show any significant differences in flow parameters between both PA’s [26]. Therefore, we analyzed left pulmonary artery (LPA) only.

All MRI scans were performed on a 1.5T whole-body MRI system in the participating centers (General Electric Signa, Philips Achieva and Siemens Avanto). The LPA was localized on a transverse steady-state free precession (SSFP) image set. On this transverse image, another localizer was planned longitudinally along the LPA. Using both these imaging planes, a flow measurement plane was planned perpendicular to the flow direction. Specific care was taken to match the imaging planes between both visits. In-plane resolution was between 1.1 and 1.6 mm, depending on patient size. Slice thickness was 6 mm. Phase-contrast measurements were obtained using unidirectional velocity encoding (VENC) of 60 cm/s. In case of aliasing, this VENC was stepwise increased with increments of 20 cm/s until aliasing disappeared. Flow was measured over 24 phases per cardiac cycle. To incorporate the effect of breathing, which is essential in the pulmonary circulation of Fontan patients, phase-contrast flow measurements were made without breath-holds and with three signal averages.

LPA contours were drawn on all phases using Flow Analysis software (Medis Medical Imaging Systems) to determine mean, maximal and minimal areas and mean, maximal and minimal flows.

Distensibility, which is the maximum change in cross-sectional area during the cardiac cycle, and pulsatility, which is the flow change during the cardiac cycle, were calculated according to the following formulas:

$${\text{distensibility}} = \frac{{\left( {{\text{maximal area}} - {\text{minimal area}}} \right)}}{\text{maximal area}}$$

$${\text{pulsatility}} = \frac{{\left( {{\text{maximal flow}} - {\text{minimal flow}}} \right)}}{\text{mean flow}}$$

### WSS Calculation

WSS was calculated by multiplying dynamic viscosity by wall shear rate (WSR). The latter is the radial gradient of the velocities at the wall. The formula of WSS is shown below:

$$\tau = \mu \left. {\frac{{{\text{d}}u}}{{{\text{d}}r}}} \right|_{r = 0}$$

where *μ* is the dynamic viscosity of blood, *u* is the blood velocity and *r* is the radius. The blood viscosity was set as 3.2 P. WSR was calculated according to van Duivenvoorden et al. [8]. In short, blood flow velocities were calculated within the LPA contours using the MRI phase-contrast images. Velocities within a distance of ≥0.5 pixels to the outer lumen border were excluded since those pixels were partially located outside of the lumen. The cross section was divided into four segments with 10° of overlap. In each segment, the velocities were projected onto one plane. Assuming blood velocity to be zero at the lumen wall, the projected velocities within 3 pixel distance inwards were fit with a second order curve and wall shear rate (WSR) per segment was calculated for each time point. WSS was calculated by multiplying WSR with the blood viscosity (3.2 P). We averaged the WSS values of four segments to obtain mean cross-sectional WSS. The analysis was performed on cardiac cycle-averaged WSS only [8].

### Healthy Controls

In healthy controls RPA was chosen for the flow and WSS analysis over the LPA since the first branching point of the LPA was closer to the bifurcation than the branching point of the RPA leading to disturbed flow signals in the LPA images. Images in these controls were acquired as part of another study protocol and were only acquired at one point in time. In this study, VENC was 150 cm/s, and images were taken during breath-hold at expiration [26].

### Statistical Analysis

Statistical analysis was performed using SPSS 21.0. Data were tested for normal distribution and expressed as mean ± standard deviation or median (interquartile range) as appropriate. Results were corrected for body surface area (BSA). Comparisons between Fontan patients and healthy controls were made using independent *T* test or Mann–Whitney *U* test as appropriate. To compare measurements between baseline and follow-up, paired samples *T* test or Wilcoxon singed-rank test were used. *p* values ≤0.05 were considered statistically significant. In order to identify predictors for WSS, linear regression analysis was performed. The relation between (non-log-transformed) age and WSS was nonlinear. In order to be able to perform multivariable regression analysis, we log-transformed age to obtain linearity. A *p* value ≤0.05 was required for a variable to be retained in the final model.

## Results

Twenty-eight patients underwent two MRI studies each with flow measurements of the LPA. In four patients, scanning planes did not properly match between both visits. These patients were therefore excluded. Another patient underwent a conversion from APC to extracardiac conduit (ECC) TCPC with concomitant PA surgery between both visits and was therefore excluded. There were no interventions during follow-up in the other patients. There were no patients with patent fenestrations at the time of both visits. There were no patients with large caliber changes or PA stenosis at the time of both visits.

The remaining 23 patients were included in the current analysis. Their characteristics are shown in Table 1. Median age at first visit was 8.1 (6.9–9.4) years and 12.3 (10.4–16.1) at the second visit. Median follow-up duration between both visits was 4.4 (4.0–5.8) years. All patients underwent the Fontan operation before the age of 7 years. An atriopulmonary connection was performed in two patients, all other patients had a TCPC (ILT *n* = 16, ECC *n* = 5). In most TCPC patients (17 out of 21), the TCPC was performed in a staged manner, preceded by a bidirectional Glenn shunt.

**Table 1:** Patient characteristics

Baseline parameters	Patients baseline (*n* = 23)	Patients follow-up (*n* = 23)	Controls (*n* = 16)
Male/female (*n*)	16/7		8/8
Age at study (years)	11.1 (9.5–16.0)	15.5 (12.5–22.7)	13.5 (12.1–15.5)
BSA (m^2^)	1.15 (1.02–1.78)	1.53 (1.28–1.77)	1.56 (1.36–1.69)
Age at Fontan completion (years)	3.3 ± 1.6		
Follow-up since Fontan completion	8.1 (6.9–9.4)	12.3 (10.4–16.1)	
Cardiac diagnosis (n)			
Tricuspid atresia	7		
Hypoplastic left heart syndrome	3		
Double inlet left ventricle	5		
Double outlet right ventricle	3		
Other	5		
Dominant ventricle (*n*)			
Left	17		
Right	6		
Impaired pulmonary blood flow pre-surgery	8		
Fontan type			
TCPC; ILT	16		
TCPC; ECC	5		
APC	2		
Pre Fontan procedures			
BT shunt	8		
PA banding	9		
Norwood	3		
Rashkind	2		
Bidirectional Glenn	17		

*BSA* body surface area, *TCPC* total cavopulmonary connection, *APC* atriopulmonary connection, *ILT* intra-atrial lateral tunnel, *ECC* extracardiac conduit, *BT shunt* Blalock-Taussig shunt, *PA banding* pulmonary artery banding

In eight patients, the initial procedure consisted of the creation of a BT shunt (Norwoord stage I excluded), and these patients were therefore considered to have an impaired pulmonary blood flow pre-surgery.

### Comparison with Controls

Table 2 shows the results of MRI measurements and WSS calculations for controls as well as for both visits for the patients. Mean flow, maximal flow and pulsatility were all significantly lower in patients than in controls, for both visits. There was a large difference in pulsatility within the patient group, between TCPC patients (range 0.22–2.88) and those with an APC (range 4.21–9.69). Mean and maximum areas were not significantly different between patients and controls, but distensibility was significantly lower in patients for both visits. Mean and maximal WSS were both significantly lower in patients, with the most distinct difference between patients and controls for maximal WSS.

**Table 2:** Comparison of flow and WSS variables

	Controls	Patients		*p* value
		Baseline	Follow-up	
HR (/min)	73 ± 11	76 ± 18	70 ± 16	0.112
Mean flow (ml/s/m^2^)	33.5 (27.2–37.5)	15.1 (14.3–19.1)*	18.7 (14.0–22.6)*	0.023^#^
Max flow (ml/s/m^2^)	101.8 (95.4–125.7)	23.8 (20.3–33.2)*	28.5 (24.4–37.5)*	0.031^#^
Pulsatility	3.32 (3.09–3.63)	1.05 (0.73–2.21)*	1.19 (0.59–1.79)*	0.605
Mean area (mm^2^/m^2^)	109 ± 22	113 ± 36	113 ± 38	0.966
Max area (mm^2^/m^2^)	143 ± 32	125 ± 40	123 ± 40	0.730
Distensibility	0.47 ± 0.13	0.17 ± 0.05*	0.15 ± 0.04*	0.167
Mean WSS ((N/m^2^)/m^2^)	0.50 (0.42–0.57)	0.36 (0.26–0.40)*	0.31 (0.26–0.40)*	0.068
Max WSS ((N/m^2^)/m^2^)	1.45 (1.24–1.59)	0.54 (0.47–0.62)*	0.47 (0.37–0.62)*	0.078

*p* values indicate differences between baseline and follow-up

* Significant difference between patients and controls

^#^Significant difference between baseline and follow-up, HR: heart rate, WSS: wall shear stress

### Comparison Between Baseline and Follow-Up

**Fig. 1:**
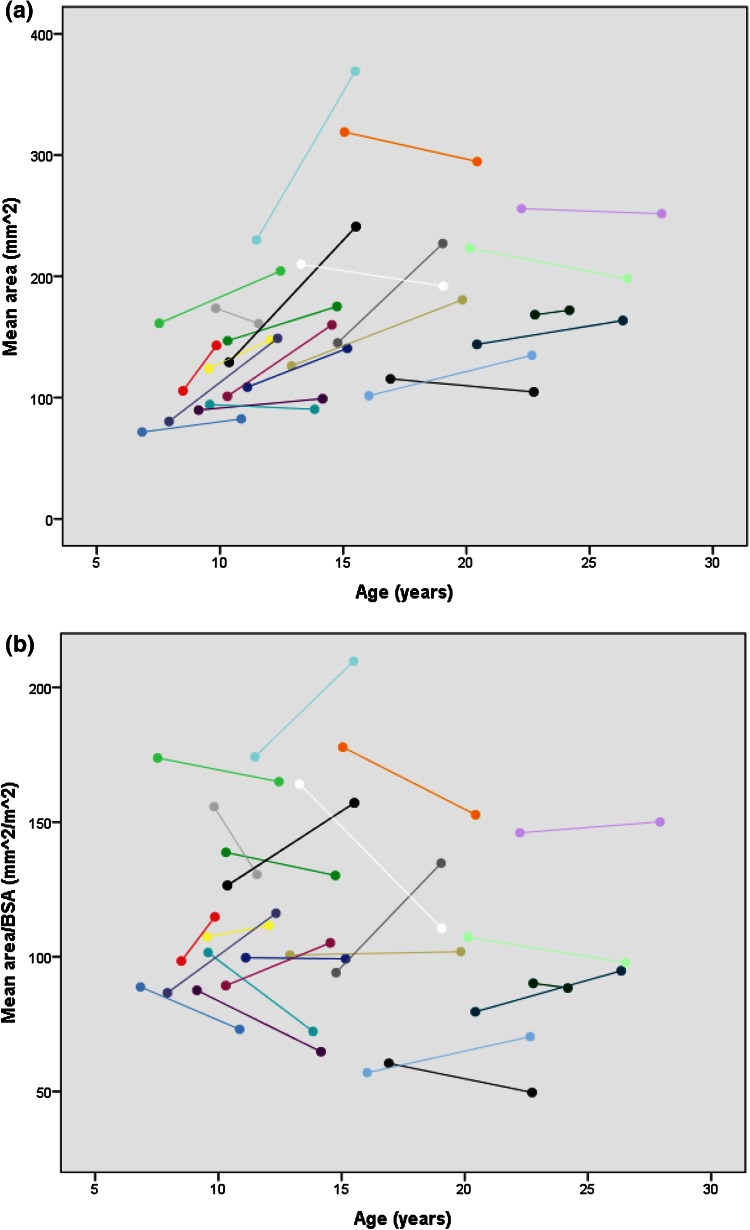
**a** Absolute mean area (mm^2^) per patient versus age (years). **b** Mean area corrected for BSA (mm^2^/m^2^) per patient versus age (years)

**Fig. 2:**
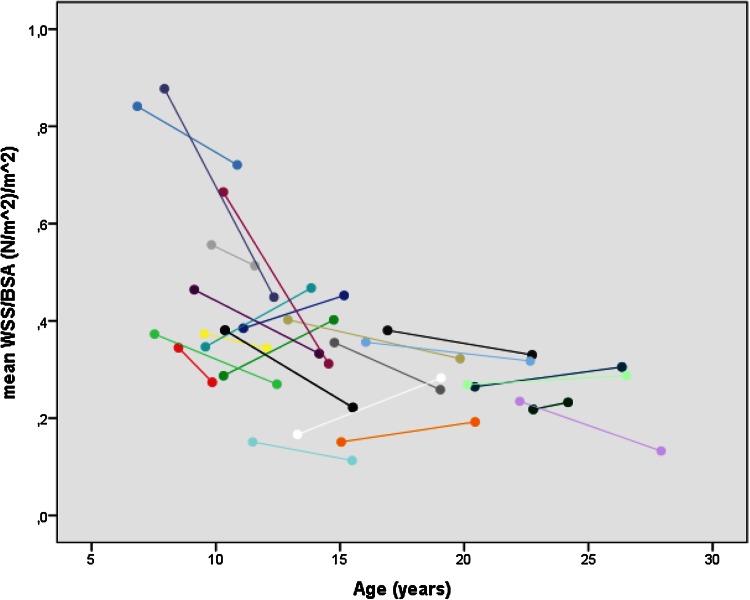
Mean WSS ((N/m^2^)/m^2^) per patient versus age (years)

The comparison between baseline and follow-up within the patients is also shown in Table 2. Mean and maximal flows (corrected for BSA) were significantly higher at follow-up. While absolute (not corrected for BSA) mean and maximal area increased significantly (mean area 156 ± 54 vs. 173 ± 57 mm^2^, *p* = 0.005; max area 186 ± 66 vs. 203 ± 65 mm^2^, *p* = 0.010), BSA-corrected mean and maximal areas did not change (Fig. 1). Distensibility and pulsatility were comparable between both visits. There was a trend toward lower mean and maximal WSS at the second visit, but the range of values was wide. Figure 2 illustrates the change of mean WSS with age. WSS is highest at a younger age, declines with increasing age and levels off at the later teen age. There was a linear relation between log-transformed age (logAge) and BSA-corrected mean WSS (*β* = −0.606, *p* < 0.001, *R*^2^ = 0.339).

### Predictors for WSS

Results of multivariable regression analysis for WSS ((N/m^2^)/m^2^) are shown in Table 3. LogAge, mean area, mean flow, pulsatility and distensibility were entered in the model. Since pulsatility was much higher in APC patients, these were excluded from the multivariable analysis. Pulsatility and distensibility did not show a significant relation with WSS. In the final model, log age, mean flow and mean area explain 85 % of the variability in WSS.

**Table 3:** Multivariable regression model (for TCPC patients only) for mean WSS ((N/m^2^)/m^2^)

Variable	β	*p* value	*R* ^2^
Constant	1.227	<0.001	0.845
Log age (log years)	−0.727	<0.001	
Mean flow (ml/s/m^2^)	0.022	<0.001	
Mean area (mm^2^/m^2^)	−0.004	<0.001	

## Discussion

This study shows that PA size in growing Fontan patients is comparable to healthy controls and increases with age, appropriate to body size. Flow in the LPA increased significantly between both visits, but was still lower than in healthy controls. Pulsatility and distensibility were impaired and did not change over time, despite increase in flow. WSS was not significantly different between baseline and follow-up. Multivariable regression analysis revealed that age, mean flow and mean area are important predictors for WSS that explain most of the variability.

### PA Size

Several studies have assessed the range of branch PA size in healthy children and the relation with age and/or body size. Using contrast-enhanced MRI, Knobel and colleagues have shown that there is a linear relation between BSA and PA size [20]. Using echocardiography, an older study showed a similar trend in younger children [28]. Our results indicate that LPA growth is proportionate to BSA in Fontan patients.

Only a few studies have investigated pulmonary artery size in Fontan patients using MRI. LPA size in our patients was comparable to that found in a previous smaller sample from our institutions [26]. Bellsham-Revell and colleagues investigated growth of the LPA between the hemi-Fontan stage and the TCPC in patients with hypoplastic left heart syndrome (HLHS) [4]. The (BSA-corrected) size of the LPA pre-TCPC in their study was comparable to the values that we have presented. In the study by Bellsham-Revell, it was found that, while there was an increase in area of the proximal LPA between stages, there was a relative decrease in size of the narrowest part of the LPA. The authors stated that the staged Fontan approach might be a risk factor itself for the narrowing of the LPA in these HLHS patients [4]. Earlier studies have shown that LPA stenosis is a well-known problem, particularly in HLHS patients [3]. In our population, there were only a few patients with HLHS. LPA areas in these patients (range 50–102 mm^2^/m^2^) were among the lowest sizes measured in our study. The previous studies and our results emphasize the need for close surveillance in this specific group.

Other studies have used X-ray catheter-assisted angiography to measure PA size, usually using the Nakata index [16, 22]. Although this makes comparison challenging, a previous study in patients with congenital heart disease has shown excellent agreement between angiography and MRI measurements of the great arteries, including branch pulmonary arteries [34].

In a recent study, Schmitt et al. [29] found a decreased Nakata index of 150 mm^2^/m^2^ in 10 Fontan patients. Converting this number into a single PA branch area as we have measured would result in a PA size of 75 mm^2^/m^2^, which is lower than we found. This difference might be explained by the fact that patients in the study by Schmitt were older and all of their patients had an unfenestrated extracardiac conduit Fontan.

Few studies have assessed PA size longitudinally after Fontan completion. One study showed that Nakata index decreased after Fontan completion, but did not affect functional outcome [1]. Another retrospective study also showed that PA growth failed to match the increase in BSA in Fontan patients [32]. A recent study by Restrepo and colleagues, using MRI-derived 3D reconstructions of 25 TCPC patients, showed a similar trend. While there was an increase in absolute vessel diameters, normalized diameters decreased significantly with age [24].

In a larger study, the same group confirmed their findings of PA size change during serial follow-up [25]. Furthermore, mean flow rates remained unchanged. Using their extensive experience in this field, the authors showed that indexed power loss in the Fontan pathway increased significantly in the cohort, particularly in patients in whom the minimum normalized left pulmonary artery decreased [25].

The finding on PA size in the recent Restrepo paper is in contrast to our measurements [25]. Possible explanations include different methods for quantification of PA size. In our study, we have used 2D MRI measurement performed in a single plane, while calculations performed by Restrepo et al. were based on a 3D reconstruction of the entire Fontan pathway. Another possible explanation is the relatively high percentage of patients with HLHS in their cohort.

### PA Function

Interestingly, there was a significant increase in flow normalized to BSA in our patients between baseline and follow-up. This is in contrast to a recent MRI study among 48 TCPC patients with follow-up duration comparable to our study, showing an increase in absolute flow over time, but no changes in flow corrected for BSA19. In the aforementioned study, it was shown that indexed power loss inside the Fontan pathway increases, despite BSA-corrected flow remaining constant [25]. It remains unclear whether and how adjustment of the pulmonary circulation to the Fontan circulation occurs. WSS may be a crucial factor in that process. WSS is known to be lower in Fontan patients as compared to healthy controls. This is clear from the mean WSS, but even more so for the maximal WSS during the cardiac cycle. This is in accordance with an earlier study [26]. Impairment of WSS in Fontan patients most likely results from a combination of reduced pulmonary flow and nearly absent pulsatility [26].

Although not significant, there was a trend toward a further decrease of WSS with longer follow-up. Plotting WSS against age showed a strong decrease of WSS at early teenage age with a stabilization after the age of 15 years. In multivariable regression analysis, (the logarithm of) age was an important predictor of WSS, independent from both body size and PA area.

Loss of pulsatility of blood flow and therefore lower mean and maximal WSS values have been associated with endothelial dysfunction of the PA’s [19, 21].

In a study including 10 young patients reduced pulsatility after bidirectional Glenn correlated to impaired endothelial relaxation [21]. Another study has demonstrated abnormal response of the PA’s to exogenous nitrous oxide (NO) in Fontan patients [18]. Supplemental NO led to a fall in PVR, suggesting an elevated basal PVR, possibly related to endothelial dysfunction [18].

A further decrease of the WSS with age could also result in deterioration of endothelial function, mediated by endothelin, a potent vasoconstrictor. A study comparing endothelin receptor expression in failed and non-failed Fontan patients has shown an overexpression of these receptors in the failed Fontan group [15].

WSS is also decreased in patients with pulmonary arterial hypertension (PAH). In contrast to Fontan patients, PAH patients have dilated pulmonary arteries, due to a longstanding elevated mean PA pressure [33]. Studies have shown a negative correlation between vessel size and WSS. It has been hypothesized that the decreased WSS in PAH patients leads to an increased arterial stiffness and reduced distensibility of the PA’s [31, 33].

More direct evidence that the reduced WSS not only influences the function of the PA’s but also has effect on the structure of the vessel wall comes from a case report of a 35-year-old Fontan patient (APC). Immunohistological analysis revealed serious changes in the composition of the (main) pulmonary artery wall [2]. There was a profound reduction of muscular component and fragmentation of elastic fibers, which might influence distensibility and the vasodilatory ability. It is likely that this is also true for younger Fontan patients, operated upon according to current techniques, but this should be further investigated.

### Clinical Implications

It has been demonstrated that exercise capacity is reduced in Fontan patients and that it reduces further with age [5]. In the current study, we have shown that WSS also decreases with age in Fontan patients. The decrease in WSS might influence endothelial function in the PA’s. In healthy subjects, there is an increase of distensibility during exercise with an increase in the release of NO [12]. This induces vasodilatation and enhances pulmonary blood flow by a decrease in pulmonary vascular resistance. In Fontan patients, there often is an increase in pulmonary vascular resistance during exercise, indicating that this mechanism is impaired. This contributes to impaired ventricular filling [10]. A previous study from our institution showed that Fontan patients are not able to increase stroke volume during exercise [27]. Other studies have shown similar results [35]. The abnormal function of the PA’s thus has direct consequences for exercise function of the patients, and may contribute to decline of exercise capacity.

Considering these observations, it is of utmost importance to be able to influence pulmonary vascular resistance in the Fontan circulation. Several studies using bosentan, an endothelin receptor antagonist, have not shown significant improvement [23, 30]. Another study using sildenafil, a phosphodiesterase-5 inhibitor, to assess the influence during exercise in Fontan patients, showed an increase in stroke volume and cardiac index and a decrease in PVR during exercise [35]. Exercise capacity improved after sildenafil administration, but mainly in those patients with a poor baseline exercise capacity. This indicates that the reduced endothelial function could be attenuated to affect exercise capacity. In another study, sildenafil was administered for a period of 6 weeks, but failed to show a significant improvement in exercise capacity [11]. It has been speculated that this result was caused by the fact that relatively fit Fontan patients were included [14]. Further studies are necessary to identify those patients that benefit the most from this potential therapy or to uncover other potential targets and means for possible therapeutical intervention.

### Limitations

Sample size was relatively small and the Fontan population is heterogeneous with respect to different cardiac diagnoses. This may be one of the reasons for differences between the outcome of this and other recent studies [25]. The subgroups were too small for group-to-group comparison.

This study assumes laminary flow in the PA’s for the calculation of WSS. Although care was taken not to measure flow too close to the caval connection point, flow disturbances may have been present, depending on the individual anatomy and intravascular flow pattern in patients.

Since measurements were performed in one of the branch pulmonary arteries, this study does not provide direct knowledge on the smaller pulmonary vasculature further downstream.

## Conclusions

WSS in Fontan patients is decreased as compared to healthy controls and decreases further with age. Pulsatility and distensibility are also significantly lower. Pulmonary artery size, however, is not significantly different from healthy controls and growth after Fontan operation remains proportionate to body size.
